# CD8 T cell exhaustion-associated LncRNA signature reveals novel molecular subtypes and immune targets in hepatocellular carcinoma

**DOI:** 10.1007/s12672-025-03675-w

**Published:** 2025-10-08

**Authors:** Jiahui Feng, Dezhong Yan, Lingling Jiang, Fengqian Song

**Affiliations:** Department of Gastroenterology, Loudi Central Hospital, Loudi, Hunan China

**Keywords:** Exhausted CD8 T cells, Hepatocellular carcinoma, Immune microenvironment, Immunotherapy, Molecular subtype

## Abstract

**Background:**

Hepatocellular carcinoma (HCC) presents challenges and opportunities for immunotherapy due to its intricate immune microenvironment. Exhausted CD8 T cells (CD8Tex) are pivotal in this context but are inadequately characterized in HCC.

**Methods:**

We conducted single-cell analysis on the GSE140228 dataset to identify key genes associated with CD8Tex in HCC. Cell communication analysis elucidated strong interactions of CD8Tex with CD8 T cells, dendritic cells (DCs), and monocytes/macrophages.

**Results:**

Pearson correlation analysis using the TCGA-LIHC dataset identified CD8Tex-associated long non-coding RNAs (lncRNAs). Utilizing univariate and multivariate Cox regression analyses, along with LASSO regression to prevent overfitting, we developed a prognostic model incorporating 5 lncRNAs. This model exhibited strong prognostic performance, and the derived risk score was validated as an independent predictor of overall survival in HCC patients. Among these lncRNAs, AL158166.1 showed the strongest correlation with CD8⁺ T cell exhaustion and was significantly associated with poor prognosis, highlighting its potential as both a biomarker and therapeutic target in HCC.

**Conclusion:**

Our study not only elucidates the role of CD8Tex cells in HCC but also proposes a novel molecular classification of the disease. This classification holds promise for guiding clinical immunotherapy and precision treatments tailored to different molecular subtypes of HCC, as identified through drug sensitivity analysis. This work provides a foundational framework for advancing clinical strategies in HCC treatment and the development of targeted therapies.

**Supplementary Information:**

The online version contains supplementary material available at 10.1007/s12672-025-03675-w.

## Introduction

Liver cancer is the third leading cause of cancer-related mortality globally, with approximately 80% of cases being hepatocellular carcinoma (HCC) [1, 2]. Emerging immunotherapies have made significant advancements in the treatment of various cancers, such as lymphoma and melanoma [3, 4]. Compared to traditional therapies like tyrosine kinase inhibitors, immune checkpoint inhibitors demonstrate superior efficacy in HCC treatment [5]. However, their effectiveness is limited by certain factors [6–9]. Among these limiting factors, the exhaustion of tumor-infiltrating lymphocytes, particularly CD8 + T cells, is a central issue [10].

T cells play a critical role in mediating protection against tumor cells but frequently undergo dysfunction and exhaustion in cancer [10]. T cell exhaustion (Tex) is a collective term encompassing all functional impairments observed in antigen-specific CD8 + T lymphocytes, originally described within the context of chronic viral infections where these cells persist but fail to clear pathogenic threats [11]. Studies indicate that blocking surface co-inhibitory receptors, such as programmed cell death protein 1 (PD-1), expressed on CD8 + Tex, can reactivate cytolytic immune responses mediated by cells to eliminate persistent viral infections [11]. Subsequently observed in cancer, CD8 + Tex exhibit similarly diminished responsiveness to tumor immunotherapy [11]. It is proposed that cells expressing PD-1 can be rescued from a non-responsive exhausted state via immune checkpoint blockade (ICB) in a straightforward unidirectional reversal [12, 13]. In cancers, this is thought to involve functionally impaired CD8 + Tex expressing high levels of PD-1, predominantly localized within the tumor microenvironment (TME) [11, 13]. Given that the liver serves not only as a vital immune organ but also as the largest metabolic organ in the body, identifying key factors driving CD8 + T cell infiltration in HCC and exploring their underlying mechanisms represent urgent scientific priorities.

Biomarkers are gaining prominence in evaluating tumor progression and prognosis in HCC [8]. Among these, long non-coding RNAs have emerged as a major area of interest due to their diverse regulatory functions and clinical relevance. Long non-coding RNAs (lncRNAs) are RNA molecules exceeding 200 nucleotides in length found ubiquitously across various organisms, traditionally not believed to encode proteins [14]. Increasing evidence implicates dysregulated expression of lncRNAs in hepatocellular carcinoma (HCC), influencing its development and progression [15], and playing critical roles in lipid metabolism [16], glucose metabolism [17], among other aspects. Moreover, recent studies indicate that lncRNAs are closely associated with the immune microenvironment of HCC and immune cell infiltration [18, 19]. However, whether lncRNAs are linked to CD8 + T cell exhaustion events in HCC and what specific roles they play there remain to be elucidated.

In our study, we analyzed key genes associated with CD8 + Tex by leveraging single-cell data from the GSE140228 dataset. Through cell-to-cell communication and immune cell function analysis, we unveiled the relevant immune functions and pathways of CD8 + Tex. By employing the TCGA-LIHC dataset, we identified CD8 + Tex-related lncRNA signatures through correlation analysis. Importantly, among the identified lncRNAs, AL158166.1 emerged as the most strongly associated with CD8⁺ Tex and adverse clinical outcomes. A prognostic model based on these CD8 + Tex-related lncRNA signatures showed a good predictive efficacy for the prognosis of HCC. Furthermore, we established a novel molecular subtyping system associated with CD8 + Tex-related lncRNAs and revealed the immunomicroenvironment heterogeneity among HCC patients of different molecular subtypes. This novel molecular subtyping of HCC can guide clinical immunotherapy for HCC patients. Our research provides a reliable theoretical foundation for the clinical treatment of HCC and the future development of targeted drugs.

## Materials and methods

### Selection of differentially expressed CD8Tex related genes in HCC

To identify gene expression patterns specific to CD8⁺ Tex cells in HCC, we performed differential gene analysis based on single-cell data. We obtained single-cell RNA data for HCC, totaling 62,530 cells, from the GSE140228 dataset on the GEO website. Next, we analyzed this data using TISH2, focusing on CD8Tex cell clusters. Using the Wilcoxon test, we found genes with significant differential expression compared to other cells, defined by log2|FoldChange| ≥ 1 and FDR < 0.05 [20].

### Cell-cell communication analysis

We analyzed single-cell RNA data from GSE140228 dataset on the GEO website. Using the TISH2 platform, we applied the Wilcoxon test to identify genes with significantly different expression in CD8Tex cell clusters compared to other cells, using criteria of log2|FoldChange| ≥ 1 and FDR < 0.05. Additionally, CellChat analysis [21] was conducted via TISH2 to study cell-cell communication based on known ligand-receptor pairs across clusters. The analysis employed the “netVisual_circle” function from the pheatmap and CellChat R packages to quantify and visualize significant interaction pairs and communication probabilities between clusters, with significance determined at a P-value threshold of 0.05.

### Functional enrichment analysis of different cell clusters

To understand the functional heterogeneity among immune cell types, we performed pathway enrichment analysis on cluster-specific gene sets. Differentially expressed genes across clusters were ranked by fold change. Enrichment analysis was conducted using Gene Ontology (GO), Kyoto Encyclopedia of Genes and Genomes (KEGG), and Gene Set Enrichment Analysis (GSEA). These tools enable pathway-level interpretation of gene expression data, identifying overrepresented biological processes and signaling cascades. Enrichment significance was set at FDR ≤ 0.05 [20].

### Collection of HCC transcriptome and clinicopathological data

To construct and validate a prognostic model, we obtained bulk RNA-seq data along with corresponding clinical annotations from HCC patients. We acquired transcriptomic data from the TCGA-LIHC dataset, including 375 HCC samples and 50 paired adjacent normal tissues. Out of these, 365 patients had complete survival data, clinical pathology details, and somatic mutation records.

### Construction and validation of prognosis-related CD8Tex related LncRNAs model in HCC

Using Pearson correlation and strict criteria (>0.4 correlation coefficient and p-value < 0.001), we identified CD8Tex-related lncRNAs. The limma package was used to analyze expression differences between hepatocellular cancer and normal tissues, identifying significantly expressed lncRNAs based on |logFC| ≥ 1 and FDR < 0.05. Heatmaps and volcano plots were created to visualize expression patterns. Based on the TCGA-LIHC dataset, we applied univariate Cox regression (FDR < 0.05) to identify CD8Tex-related lncRNAs associated with HCC prognosis. To avoid overfitting, we performed LASSO regression using the R package glmSparseNet [19]. This process led to the establishment of an HCC prognosis model comprising 11 biomarkers (BRGs). For each TCGA-LIHC sample, we calculated risk scores. A 1:1 cutoff was used to stratify the TCGA-LIHC samples into low-risk and high-risk groups. Through Kaplan-Meier survival and ROC analyses in R (survival package [19]; timeROC package [19]), we assessed the predictive accuracy of the model for HCC prognosis.

### Functional enrichment analysis of different risk score groups

To explore the molecular mechanisms underlying different prognostic risk levels, we conducted enrichment analysis between high- and low-risk groups. For differential gene analysis between high-risk and low-risk groups in TCGA-LIHC, we utilized the limma package in R with criteria of |logFC| >1 and FDR < 0.05. Subsequently, KEGG and GO enrichment analyses were performed using the clusterProfiler package in R to investigate molecular mechanisms associated with HCC risk scores. Additionally, GSEA was employed to evaluate biological functional changes between low and high-risk subgroups, setting thresholds at |NES| >1 and FDR < 0.05 [19].

### Assessing the clinical relevance of CD8Tex related LncRNAs models

To establish the risk score as an independent prognostic factor for HCC, we performed univariate Cox regression analysis (FDR < 0.05) and multivariate Cox regression analysis (FDR < 0.05) using the survival package in R. Subsequently, ROC curves were generated using the survminer [19] and timeROC [19] packages in R to compare the predictive performance of the risk score against other clinicopathological factors for HCC prognosis. Visualization of the correlation between the risk score and other clinicopathological factors of HCC was conducted using the ComplexHeatmap and reshape2 packages in R.

### Evaluate the ability of the CD8Tex related LncRNAs model to characterize the immune microenvironment of HCC

Based on the TCGA-LIHC dataset, we utilized various algorithms including TIMER (Tumor Immune Estimation Resource) [18], ssGSEA (single-sample Gene Set Enrichment Analysis) [18], MCP (Microenvironment Cell Population) Counter [18], QUANTISEQ (QUANTItative Immune Single-cell Expression) [18], Estimation [18], CIBERSORT (Cell-type Identification By Estimating Relative Subsets Of RNA Transcripts) [18], and EPIC (Evaluating the Proportion of Immune Cells) [18]. These algorithms were employed to quantify the relative levels of tumor-infiltrating immune cells in TCGA-LIHC samples. Specifically, ssGSEA and CIBERSORT were used to estimate immune cell subpopulation abundance, and ssGSEA was additionally used to quantify immune functional status across samples.

### Assessment of response to immunotherapy

Based on the TCGA-LIHC dataset, we utilized the R packages limma and reshape2 [18] to analyze the differential expression of immune checkpoint genes among different risk score groups. Our analysis identified immune checkpoint genes that showed significantly altered expression levels across these groups. These findings suggest that higher expression levels of these genes may correlate with increased efficacy of immune checkpoint inhibitors in the context of HCC.

### Identification of novel molecular subtypes of HCC

Based on the TCGA-LIHC dataset, we employed the R package ConsensusClusterPlus [18] to conduct unsupervised consensus clustering, aiming to identify novel molecular subtypes of HCC. This analysis yielded several key outputs: s.

Consensus Matrix (CM): This matrix illustrates how frequently sample pairs are grouped together across iterations, providing a quantitative measure of clustering stability and robustness.

Cumulative Distribution Function (CDF) plot: This plot helps determine the optimal number of clusters by showing the stability of clustering outcomes across different cluster numbers.

Consensus heatmap: Visual representation of the consensus matrix, aiding in the interpretation of clustering results by highlighting patterns of sample grouping.

These tools collectively support the identification of distinct molecular subtypes within HCC based on underlying genetic and molecular characteristics. This approach enhances our understanding of the heterogeneity of HCC and can potentially guide personalized treatment strategies tailored to specific molecular subtypes.

### Statistical analysis

Based on the TCGA-LIHC dataset, all statistical analyses were conducted using R version 4.3.0 [22]. Here’s a summary of the statistical methods employed:

**Kruskal-Wallis Test**: Used to examine variances in immune scores, immune checkpoint gene expressions, and drug sensitivities across different clusters identified by ConsensusClusterPlus. This non-parametric test assesses whether there are statistically significant differences between groups.
**Log-Rank Test**: Employed from the R survival package for Kaplan-Meier (KM) analysis to compare the differences in patient survival rates between two risk groups defined by immune scores or other clinical variables. This test helps determine whether there is a significant difference in survival distributions between the groups.
**Statistical Significance**: Throughout the analyses, two-sided tests were used, where p-values less than 0.05 were considered statistically significant. The levels of statistical significance were denoted with asterisks: * (*p* < 0.05), ** (*p* < 0.01), and *** (*p* < 0.001), indicating the strength of significance observed in the results.

These statistical approaches were crucial in evaluating the relationships between molecular subtypes, immune profiles, and clinical outcomes in hepatocellular carcinoma (HCC), providing valuable insights into potential therapeutic strategies and prognostic markers.

## Results

### CD8Tex profile in HCC

**Fig. 1:**
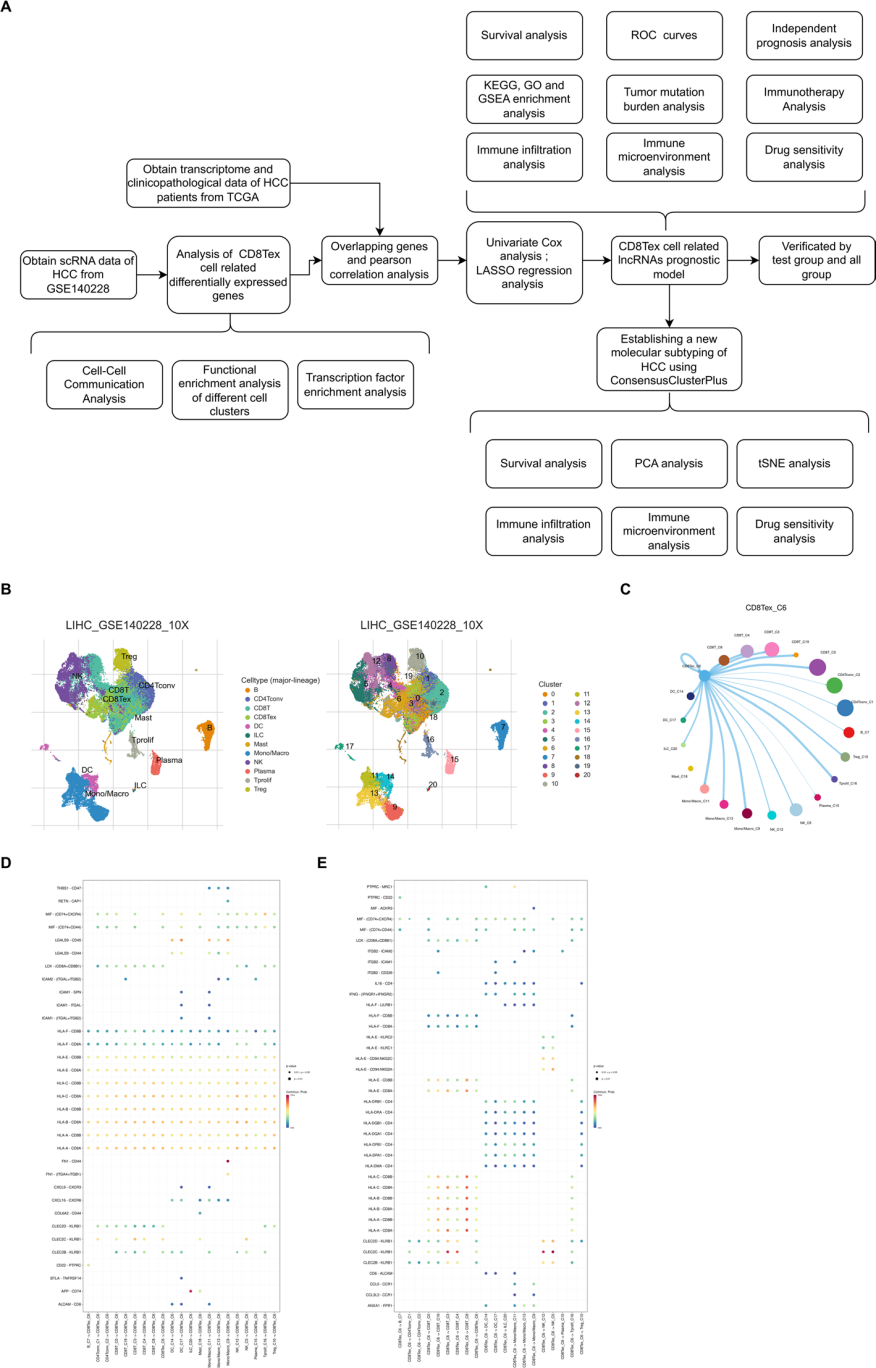
CD8Tex cell-to-immune cell communication in HCC. **A** Detailed flowchart illustrates the identification and validation of the prognostic model associated with CD8Tex related genes. **B** The cellular map illustrates the distribution and abundance of various cell subgroups within HCC. **C** Using CellChat, the interaction probability between specific cell groups and other cell groups is depicted. **D** Using CellChat, the interaction probability is delineated between CD8Tex cells as donors and specific gene pairs of other cells. **E** Using CellChat, the interaction probability is delineated between CD8Tex cells as recipients and specific gene pairs of other cells

Following the workflow depicted in Fig. 1A, we initially analyzed the hepatocellular carcinoma (HCC) single-cell dataset GSE140228 and identified the CD8Tex cluster (Fig. 1B). In the tumor microenvironment, cellular interactions not only regulate cell functions but also influence the surrounding immune milieu, thereby impacting tumor progression [23]. Using the CellChat algorithm, we identified strong interactions of CD8Tex with CD8T cells, dendritic cells (DCs), and monocytes/macrophages, while interactions with CD4T cells and B cells were relatively weaker (Fig. 1C). Subsequently, we analyzed the gene pairs involved in interactions between CD8Tex and other cells. The results indicated that CD8Tex, as a receptor, is regulated by families such as HLA and CLEC2D from other cells (Fig. 1D). Conversely, when CD8Tex acts as a sender, it regulates other cells through families like PTPR and ITGB. These findings underscore the significant role of CD8Tex in mediating intercellular communication within the immune landscape of HCC.

**Fig. 2:**
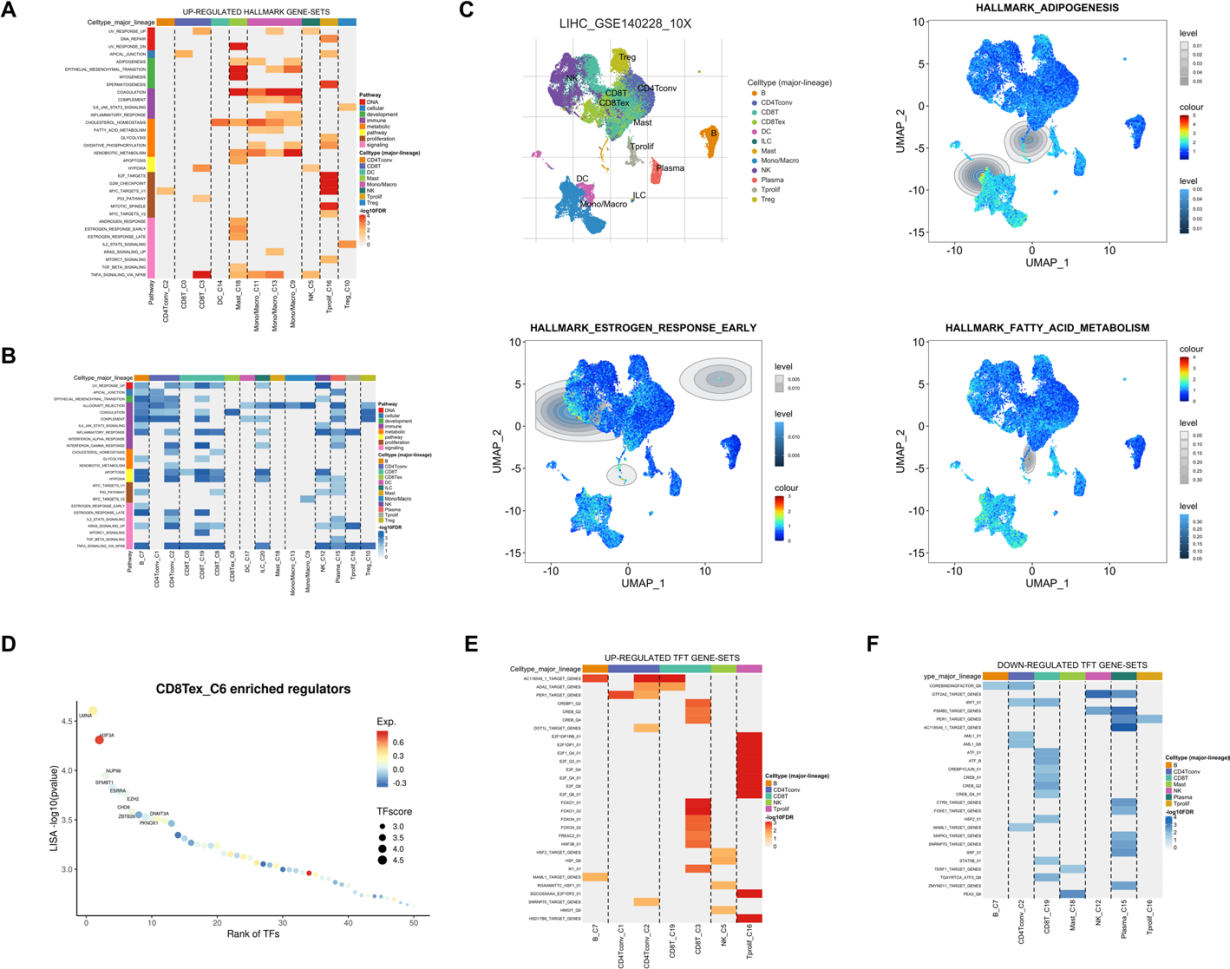
Analysis of CD8Tex cell-related immune signatures in HCC. **A** The heatmap exhibits hallmark enrichment pathways positively regulated by each cell subgroup. **B** The heatmap exhibits hallmark enrichment pathways negatively regulated by each cell subgroup. **C** Heatmap showing the cellular distribution of expression levels of different gene sets. **D** The dot plot displays significantly expressed transcription factors in CD8Tex cells of HCC. **E** The heatmap illustrates key transcription factors up expressed in various cells within HCC. **F** The heatmap illustrates key transcription factors down expressed in various cells within HCC

Next, we sought to identify gene sets enriched in CD8Tex to elucidate its cellular functions. We found no upregulated gene sets in CD8Tex, but observed significant downregulation of COAGULATION, suggesting potential immunological roles of CD8Tex (Fig. 2A, B). Furthermore, we noted substantial inhibition of crucial immune-related pathways such as HALLMARK-ADIPOGENESIS, HALLMARK-ESTROGEN RESPONSE EARLY, and HALLMARK-FATTY ACID METABOLISM in CD8Tex (Fig. 2C). Given the well-established functions of most transcription factors in HCC, we analyzed those enriched in CD8Tex to uncover their roles. We identified significant enrichment of LMNA and H3F3A in CD8Tex (Fig. 2D). However, no enriched transcription factor sets were found in CD8Tex, suggesting it may exert regulatory effects through individual transcription factors such as LMNA and H3F3A (Fig. 2E, F).

### Identification of CD8Tex-related LncRNA and establishment of a prognostic model

**Fig. 3:**
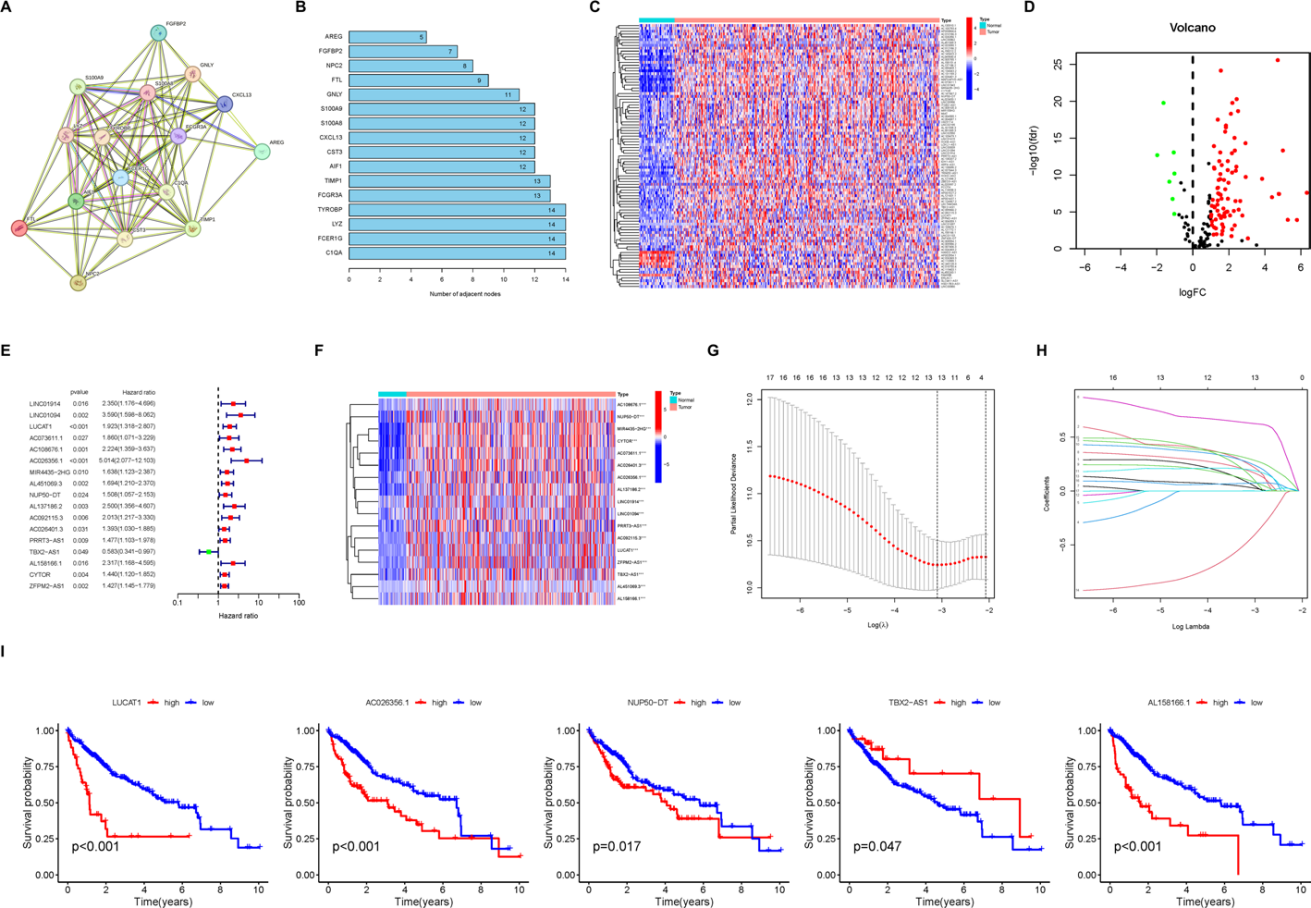
Establishment of prognostic model of CD8Tex cell-related lncRNAs in HCC. **A** Protein-protein interaction network of key genes related to CD8Tex cells. **B** Bar graph showing the number of nodes for each key gene in the protein-protein interaction network. **C** The heatmap demonstrates the expression levels of CD8Tex cell-related lncRNAs in both hepatocellular carcinoma (HCC) and paired adjacent non-tumor samples. **D** Volcano plot illustrating differentially expressed CD8Tex cell-related lncRNAs in HCC. **E** The forest plot presents CD8Tex cell-related lncRNAs associated with HCC prognosis. Green indicates hazard ratio < 1; red indicates hazard ratio > 1. **F** The heatmap illustrates the expression levels of CD8Tex cell-related lncRNAs associated with HCC prognosis in both HCC and paired adjacent non-tumor samples. **G** Cross-validation curve of LASSO regression analysis demonstrating the model’s fitting effect with varying numbers of CD8Tex cell-related lncRNAs. **H** LASSO coefficient path plot showing lambda values when incorporating different numbers of CD8Tex cell-related lncRNAs into the model. **I** Survival analysis depicting the relationship between the expression levels of five CD8Tex cell-related lncRNAs included in the prognostic model and the prognosis of HCC patients

**Fig. 4:**
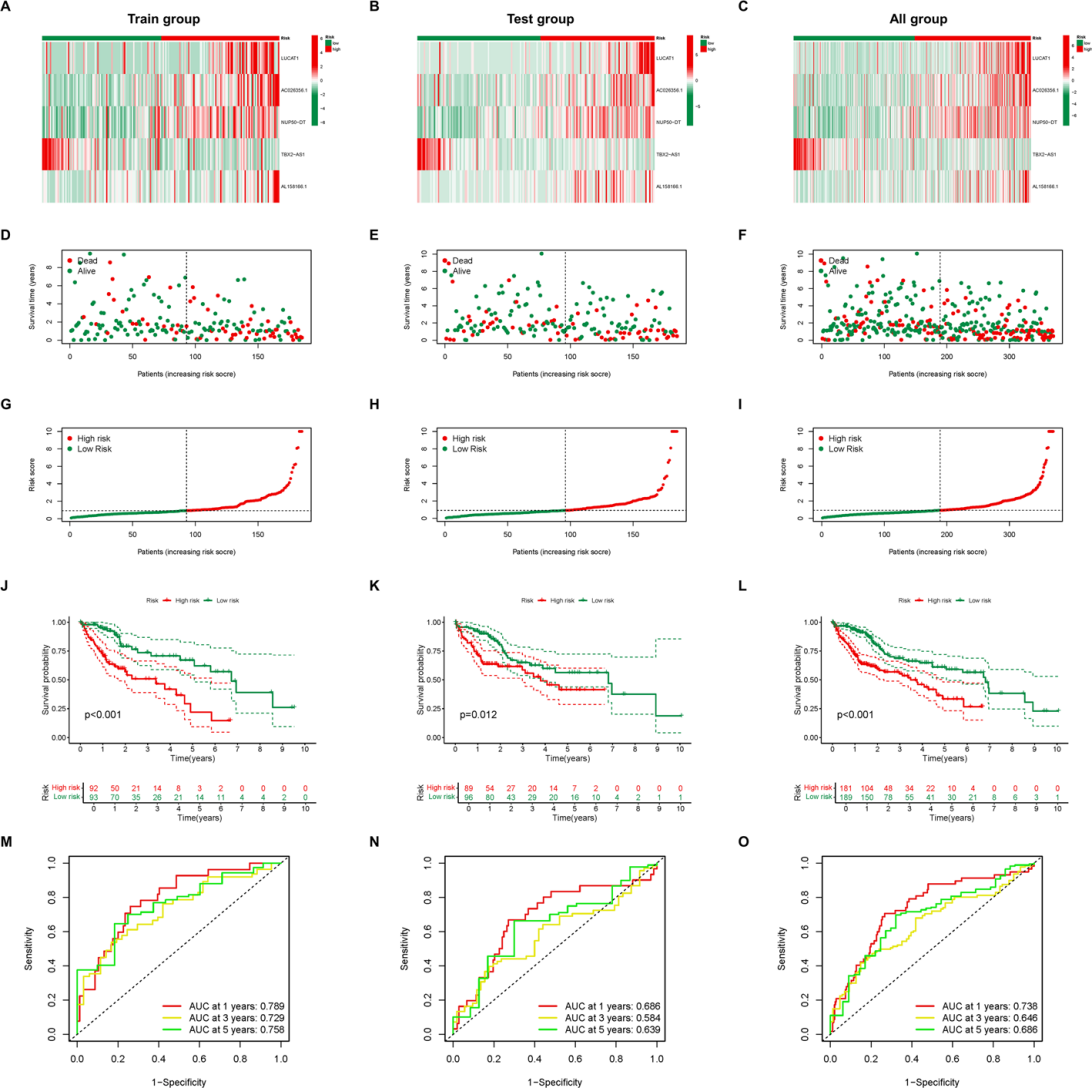
Validation of CD8Tex cell-related lncRNAs prognostic model in HCC. **A**–**C** The heatmap displays the expression levels of the 5 CD8Tex cell-related lncRNAs included in the prognostic model in train group (**A**), test group (**B**), and all group (**C**). **D**–**F** The dot plots show the distribution of the survival status of HCC patients with different risk scores in train group (**D**), test group (**E**), and all group (**F**). **G**-**I** The risk curves depict the distribution of HCC patients with different risk scores in train group (**G**), test group (**H**), and all group (**I**). **J**–**L** The survival curves illustrate the prognosis of HCC patients with different risk scores in train group (**J**), test group (**K**), and all group (**L**). **M**-**O** The ROC curves demonstrate the predictive efficacy of the CD8Tex cell-related lncRNAs prognostic model in train group (**M**), test group (**N**), and all group (**O**)

To identify genes specifically expressed in CD8Tex, we applied a threshold of |Fold Change| ≥ 2 and FDR < 0.05 to detect differentially expressed genes, identifying 1 upregulated and 21 downregulated genes (Table S1). Subsequently, we utilized the STRING database for further analysis to pinpoint 16 core genes (Fig. 3A, B). We obtained data from TCGA-LIHC, and using Pearson correlation analysis (> 0.4 correlation coefficient and p-value < 0.001), we screened for 184 lncRNAs highly associated with these 16 core genes (Table S2). Out of these, through differential analysis, only 93 lncRNAs were found to be differentially expressed in HCC, with 7 downregulated and 86 upregulated lncRNAs (Fig. 3C-D, Table S3). We divided TCGA-LIHC data into a training and validation set in equal proportions. Following univariate COX regression analysis, we identified 17 lncRNAs related to the prognosis of HCC (Fig. 3E), and their expression levels in HCC samples were displayed (Fig. 3F). We excluded model overfitting through LASSO regression analysis (Fig. 2G, H), and through multivariate COX regression analysis, we determined 5 lncRNAs for inclusion in the prognostic model along with their risk score weights (Fig. 3I, Table S4). We found that the expression patterns of the 5 lncRNAs were consistent across the training, validation, and total sets (Fig. 4A–C), and the distribution of patients with different survival statuses was consistent (Fig. 4D–F), with risk score curves being similar (Fig. 4G–I). This suggests that there is no significant selection bias between our training and validation sets. Subsequently, we attempted to validate the predictive performance of the 5-lncRNA prognostic model. By plotting survival curves, we found that patients in the high-risk group had worse prognoses in the training, validation, and total sets (Fig. 4J–L). Moreover, ROC curves demonstrated that the 5-lncRNA prognostic model showed good predictive efficacy for the 1-, 3-, and 5-year survival rates of HCC patients in the training, validation, and total sets (Fig. 4M–O).

### Clinical relevance of risk score models

**Fig. 5:**
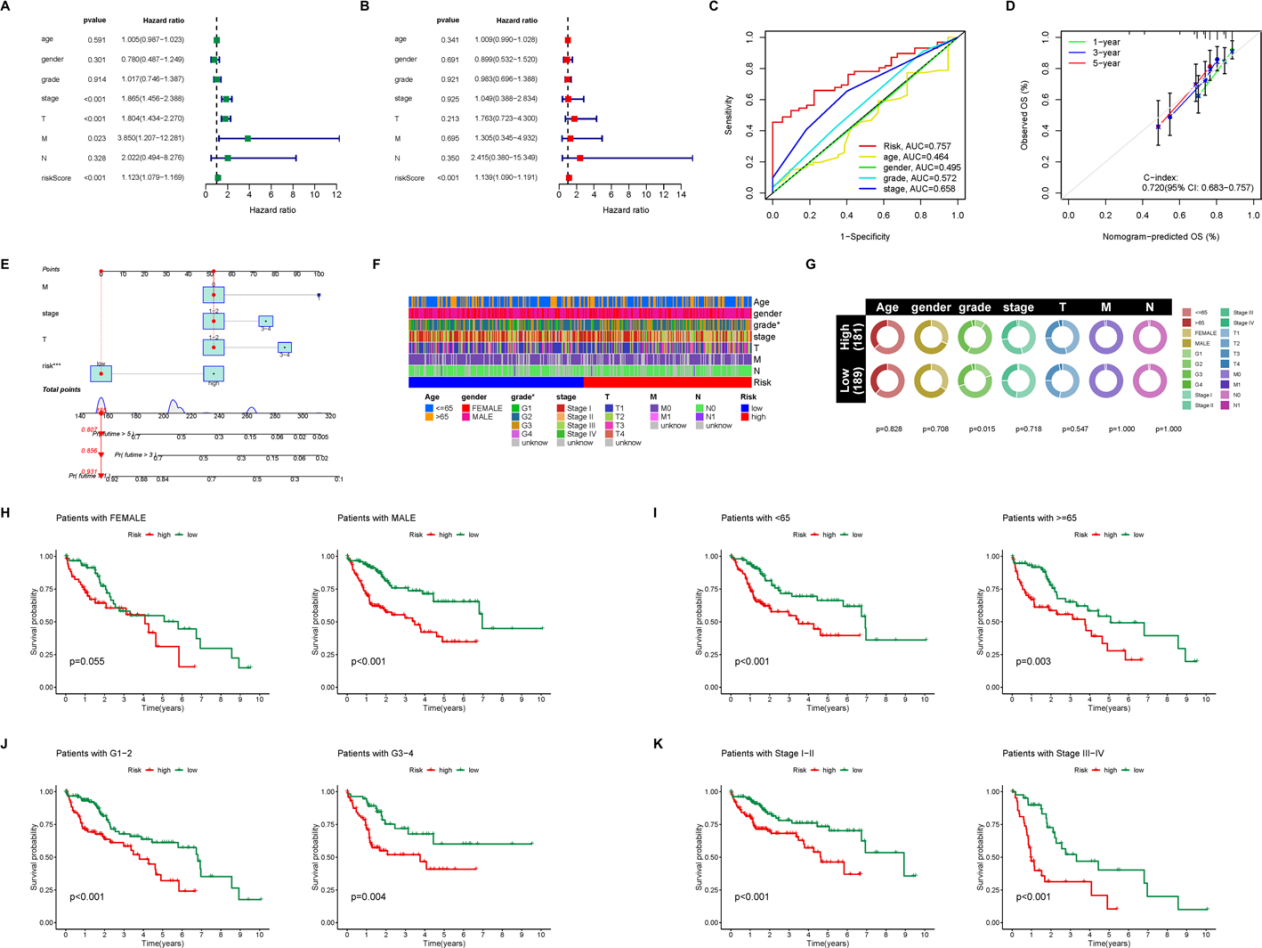
Clinical correlation analysis of CD8Tex cell-related lncRNAs prognostic model in HCC. **A** Single-factor COX regression analysis shows the association between riskscore and other clinicopathological factors of HCC with prognosis. **B** Multi-factor COX regression analysis demonstrates whether riskscore and other clinicopathological factors of HCC can serve as independent prognostic factors for HCC. **C** ROC curve illustrates the effectiveness of riskscore and other clinicopathological factors of HCC in predicting the prognosis of HCC patients. **D** Nomogram establishes specific scoring criteria for evaluating HCC prognosis. **E** The C-index curve evaluates the effectiveness of the forest plot. **F** The heatmap displays the distribution of various clinical and pathological statuses of HCC patients in different risk score groups. **G** Bubble plot demonstrates the proportions of various clinical and pathological statuses among HCC patients in different risk score groups and their correlation with riskscore. **H**–**K** Survival analysis shows the ability of the CD8Tex cell-related lncRNAs prognostic model to predict the prognosis of HCC patients in various clinical subgroups

To evaluate the clinical applicability of the 5-lncRNA prognostic model, we performed both univariate (FDR < 0.05) and multivariate COX regression analyses (FDR < 0.05) to assess the association between the risk score and other critical clinical factors. The results demonstrated that the risk score was significantly associated with poor prognosis in HCC patients and was identified as the only independent prognostic factor (Fig. 5A, B). Additionally, ROC curve analysis revealed that the risk score exhibited superior predictive performance compared to traditional clinical staging factors such as grade and stage (Fig. 5C). We then constructed a nomogram incorporating the risk score and other clinical factors to provide a tool for clinicians to evaluate HCC patients, and this model displayed a high degree of accuracy (Fig. 5D, E). We also examined the correlation between the risk score and clinical factors, revealing a strong association between the risk score and high grade in HCC patients (Fig. 5F, G). Furthermore, we assessed the efficacy of the 5-lncRNA prognostic model across various clinical subgroups. Our analysis showed that the risk score effectively predicts prognosis in HCC patients regardless of age, grade, or stage, including for male patients (Fig. 5H–K). Although there was a trend suggesting a high-risk score correlates with poor prognosis in female patients, it did not reach statistical significance (*p* = 0.055) (Fig. 5H).

### Characterization of enriched pathways in different risk score groups

**Fig. 6:**
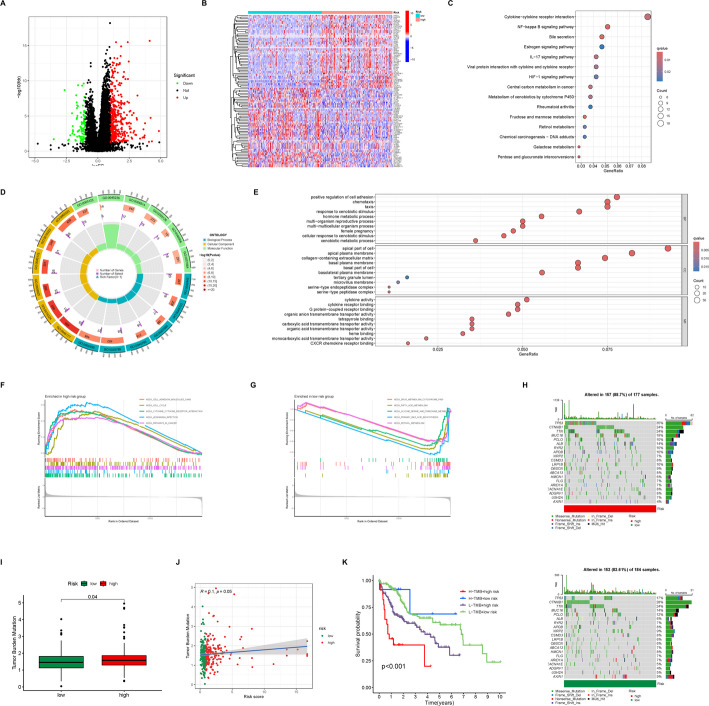
Functional enrichment analysis of different risk score groups. **A** The volcano plot illustrates the differentially expressed genes across different risk score groups. Red indicates Foldchange > 2, FDR < 0.05; green indicates Foldchange < -2, FDR < 0.05. **B** The heatmap displays the expression levels of the top fifty differentially expressed genes across different risk score groups in HCC samples. **C** The bubble plot presents the KEGG pathway enrichment analysis results across different risk score groups. **D** The bubble plot demonstrates the GO enrichment analysis pathways and proportions across different risk score groups. **E** The bubble plot exhibits the GO enrichment analysis pathways, the number of genes, and their statistical significance across different risk score groups. **F** GSEA analysis reveals the key pathways enriched in HCC patients with high risk scores. **G** GSEA analysis unveils the key pathways enriched in HCC patients with low risk scores. **H** Somatic mutation data analysis displays the mutation rates of key genes in HCC patients with different risk scores. **I** Somatic mutation data analysis displays differences in tumor mutation burden among HCC patients with different risk scores. **J** Correlation analysis shows the association between risk scores and tumor mutation burden in HCC patients. **K** Survival analysis demonstrates the impact of tumor mutation burden combined with riskscore groups on the prognosis of HCC patients

To investigate the potential regulatory roles of CD8Tex-related genes and lncRNAs in HCC prognosis, we conducted gene enrichment analyses across different risk score groups. Differential analysis identified 84 downregulated and 374 upregulated genes in the high-risk group (Fig. 6A, B). KEGG enrichment analysis highlighted enrichment in crucial immune signaling pathways such as Cytokine-cytokine receptor interaction, NF-kappa B signaling pathway, IL-17 signaling pathway, and HIF-1 signaling pathway, as well as in metabolic pathways like Central carbon metabolism in cancer, Fructose and mannose metabolism, and Galactose metabolism (Fig. 6C). GO enrichment analysis revealed enrichment in immune response processes such as CXCR chemokine receptor binding, G protein-coupled receptor binding, cytokine receptor binding, and positive regulation of cell adhesion. Additionally, metabolic processes such as hormone metabolic process, xenobiotic metabolic process, and serine-type endopeptidase complex were enriched (Fig. 6D, E). Gene set enrichment analysis (GSEA) showed that patients in the high-risk group were enriched in pathways such as KEGG_CELL_ADHESION_MOLECULES_CAMS, KEGG_CELL_CYCLE, KEGG_CYTOKINE_CYTOKINE_RECEPTOR_INTERACTION, KEGG_LEISHMANIA_INFECTION, and KEGG_PATHWAYS_IN_CANCER, indicating activation of immune signaling and cancer-related pathways (Fig. 6F). Conversely, patients in the low-risk group showed enrichment in pathways like KEGG_DRUG_METABOLISM_CYTOCHROME_P450, KEGG_FATTY_ACID_METABOLISM, KEGG_GLYCINE_SERINE_AND_THREONINE_METABOLISM, KEGG_PRIMARY_BILE_ACID_BIOSYNTHESIS, and KEGG_RETINOL_METABOLISM, suggesting comprehensive suppression of immune metabolic pathways (Fig. 6G). These findings suggest that CD8Tex and CD8Tex-related lncRNAs may primarily regulate immune responses and immune metabolism in HCC, contributing to the understanding of poor prognosis in high-risk patients.

To determine if the function of CD8Tex cells in HCC is associated with mutations in key genes related to tumor progression and prognosis, we conducted further analysis. We observed a significantly higher mutation rate in critical genes such as TP53 (34% vs. 17%) among patients in the high-risk score group (Fig. 6H). Additionally, these patients exhibited a higher tumor mutation burden (TMB) (Fig. 6I), which increased with higher risk scores (Fig. 6J). We attempted to assess prognosis in HCC patients by combining TMB and risk scores. Our findings indicated that patients with high TMB and high risk scores had the poorest prognosis. Conversely, those with low TMB and low risk scores had the best prognosis. Patients with either high TMB or high risk scores alone fell between these two extremes (Fig. 6K). These results suggest that the function of CD8Tex cells in HCC may be associated with somatic mutations, and integrating TMB with the prognostic model based on CD8Tex-related lncRNAs can enhance the prediction of prognosis in HCC patients.

### Analysis of immune microenvironment in patients with different risk score groups

**Fig. 7:**
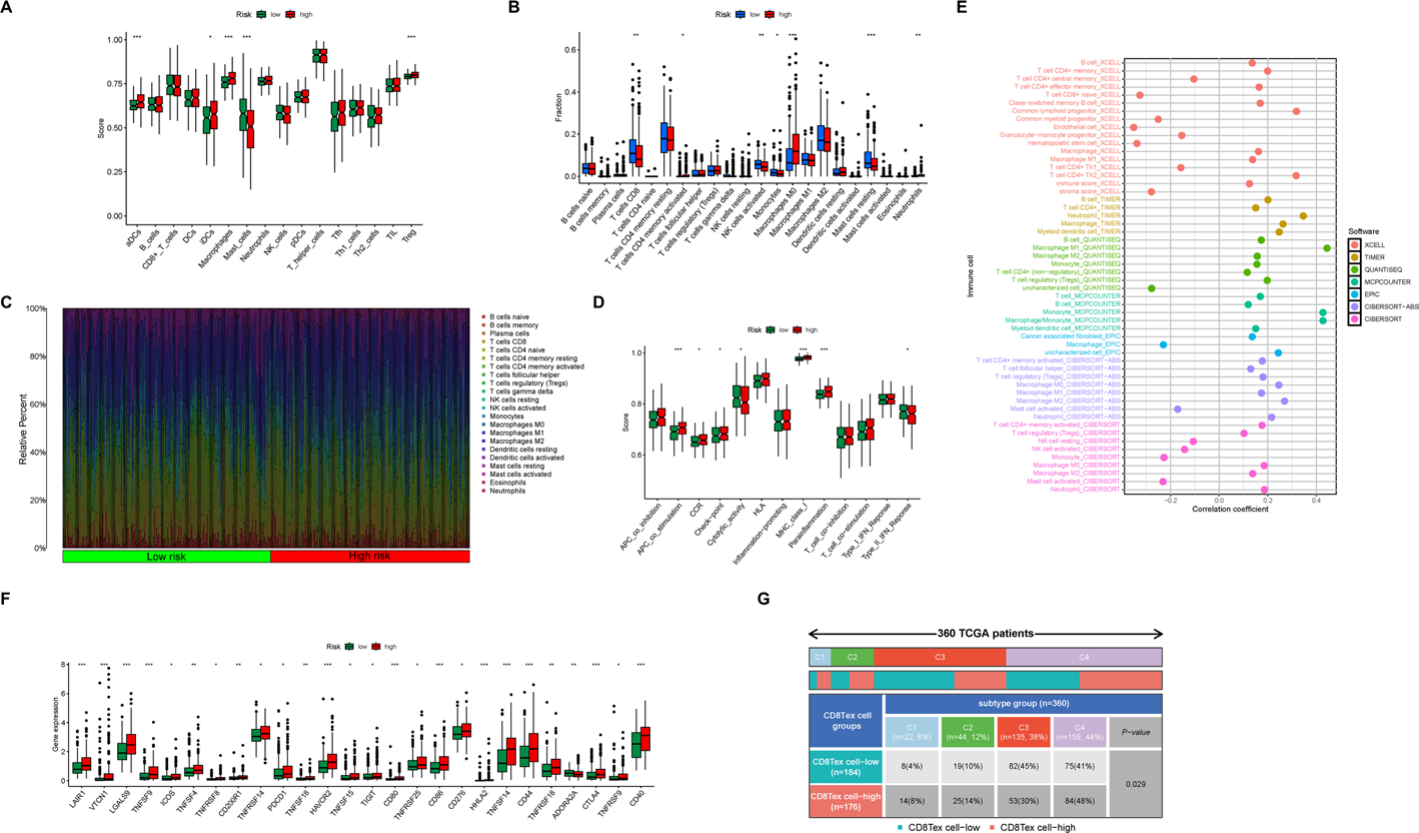
Assessment of the immune microenvironment in different risk score groups. **A** The box plot shows the abundance of immune cells across different risk score groups calculated based on the ssGSEA algorithm. **B** The box plot exhibits the abundance of immune cells across different risk score groups calculated based on the CIBERSORT algorithm. **C** The heatmap displays the distribution and proportions of immune cells across different risk score groups calculated based on the CIBERSORT algorithm. **D** The box plot demonstrates the immune functional status across different risk score groups calculated based on the ssGSEA algorithm. **E** The bubble plot demonstrates the correlation between riskscore and immune cell infiltration within HCC samples. **F** The box plot showcases the expression of immune checkpoint genes across different risk score groups. **G** The association between TCGA immune subtypes and CD8Tex cell-related lncRNAs prognostic subtypes. *p < 0.05, **p < 0.01, ***p < 0.001

To further confirm the potential role of CD8Tex and CD8Tex-related genes, we analyzed the immune microenvironment across different risk score groups. Our results showed increased infiltration of immune cells such as aDCs, iDCs, and Tregs in the high-risk group, with a decrease in Mast_cells infiltration (Fig. 7A). Using the CIBERSORT algorithm to further subclassify immune cells, we found significantly reduced infiltration of T cells CD8, NK cells activated, and Mast cells resting in the high-risk group. Conversely, there was a notable increase in Macrophages M0 and Neutrophils infiltration (Fig. 7B, C). Regarding immune function, the high-risk score group exhibited activation of immune stress and inflammatory response-related functions like APC_co_stimulation, CCR, Check-point, MHC_class_I, and Parainflammation. However, functions such as Cytolytic_activity and Type_II_IFN_Response, which are related to phagocytosis and immune surveillance, were suppressed (Fig. 7D). Correlation analysis among immune cells revealed a positive association between high-risk scores and cell fractions of Common lymphoid progenitor, T cell CD4 + Th2, Neutrophil, Macrophage M1, Monocyte, and Macrophage/Monocyte. Conversely, there was a negative correlation with cell fractions of T cell CD8 + naive, Common myeloid progenitor, Endothelial cell, and Hematopoietic stem cell (Fig. 7E). Immune checkpoint inhibitors have become a conventional treatment for HCC. Upon examining the expression of immune checkpoint genes in different risk score groups, we observed elevated expression of almost all immune checkpoint genes (except ADORA2A) in the high-risk group, suggesting that these patients could benefit from immune checkpoint inhibitors (Fig. 7F). Drug sensitivity analysis indicated that patients in the high-risk group might benefit from targeted drugs like ABT737, while those in the low-risk group might benefit from drugs like Sorafenib (Figure S1). Additionally, when comparing the prognostic typing based on CD8Tex-related lncRNAs with the official TCGA immune typing, our results showed that the prognostic typing could distinguish immune subtypes. Specifically, there was a significant increase in the number of patients with immune C4 (Inflammatory type) and a decrease in patients with immune C3 (Lymphocyte Deplete type) in the high-risk score group (Fig. 7G).

### A novel molecular classification of HCC based on CD8Tex-related LncRNAs

**Fig. 8:**
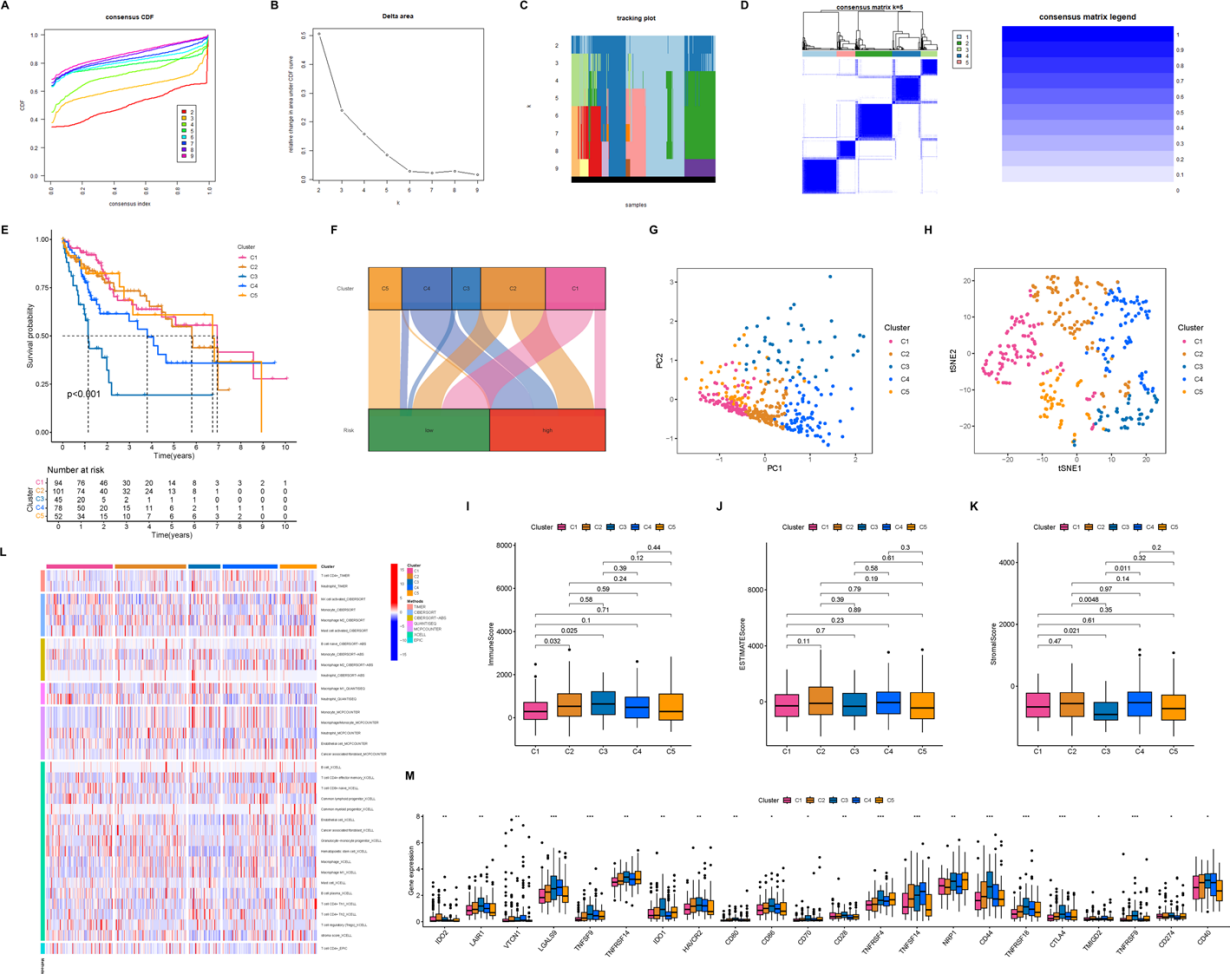
Novel molecular subtyping for identification of HCC based on CD8Tex cell-related lncRNAs. **A** Cumulative Distribution Function (CDF) curves for different numbers of classifications, where different curves represent the stability of models at different K values. **B** Distribution of samples across different numbers of classifications. **C** The heatmap displays the distribution of HCC patients under different K values. **D** Consistency clustering plot displays the clustering results when K equals 5. **E** Survival curves illustrate the prognosis of HCC patients with different molecular subtypes. **F** Sankey diagram shows the correspondence between different molecular subtypes of HCC patients and different risk score groups. **G** PCA analysis shows the distribution of samples across different HCC molecular subtypes. **H** t-SNE analysis shows the distribution of samples across different HCC molecular subtypes. **I** The box plot displays the ImmuneScore across different HCC molecular subtypes. **J** The box plot demonstrates the ESTIMATE score across different HCC molecular subtypes. **K** The box plot showcases the StromalScore across different HCC molecular subtypes. **L** The heatmap displays the immune cell infiltration status across different HCC molecular subtypes based on different algorithms. **M** The box plot demonstrates the expression of immune checkpoint genes across different HCC molecular subtypes. *p < 0.05, **p < 0.01, ***p < 0.001

Identifying accurate molecular subtypes of HCC facilitates personalized precision therapy [18]. Utilizing all five lncRNAs from the CD8Tex-related prognostic model, we employed Consensus Cluster Plus to classify HCC patients into distinct molecular subtypes. Based on Delta area and CDF curves, optimal clustering stability was observed at k = 5 (Fig. 8A-B). Sample distributions across different k values were depicted (Fig. 8C). The consistency matrix heatmap indicated relative stability with a blue shade predominating at k = 5 (Fig. 8D). Survival analysis revealed the poorest prognosis in the C3 group, whereas C1, C2, and C5 exhibited favorable prognostic outcomes (with no significant inter-group differences), and the C4 group displayed intermediate prognosis (Fig. 8E). Sankey diagrams illustrated varying proportions of high-risk and low-risk patients within C1 and C2, predominantly high-risk patients in C3 and C4, and exclusively low-risk patients in C5 (Fig. 8F). PCA (Fig. 8G) and tSNE analyses (Fig. 8H) demonstrated robust differentiation of HCC patients based on distinct molecular characteristics in the novel subtype classification. Immune microenvironment analysis indicated that the C3 group had the lowest StromalScore, while no significant differences were observed among groups in terms of ImmuneScore and ESTIMATEScore (Fig. 8I-K). Regarding immune cell infiltration, the C3 group showed minimal immune cell infiltration compared to other groups (Fig. 8L). Analysis of immune checkpoint genes suggested potential benefits from CD274 inhibitors for C1, IDO2 inhibitors for C2, LAIR1 inhibitors for C3, VTCN1 inhibitors for C4, and NRP1 inhibitors for C5 (Fig. 8M), guiding the use of immune checkpoint inhibitors. Furthermore, drug sensitivity analysis provided insights into potential benefits from targeted therapies such as Elephantin for C1, ERK_6604 for C2, XAV939 for C3, VX-11 for C4, and MG-132 for C5 (Figure S2), supporting clinical personalized treatment strategies.

### Functional validation of CD8⁺ Tex-associated LncRNA AL158166.1 in HCC

**Fig. 9:**
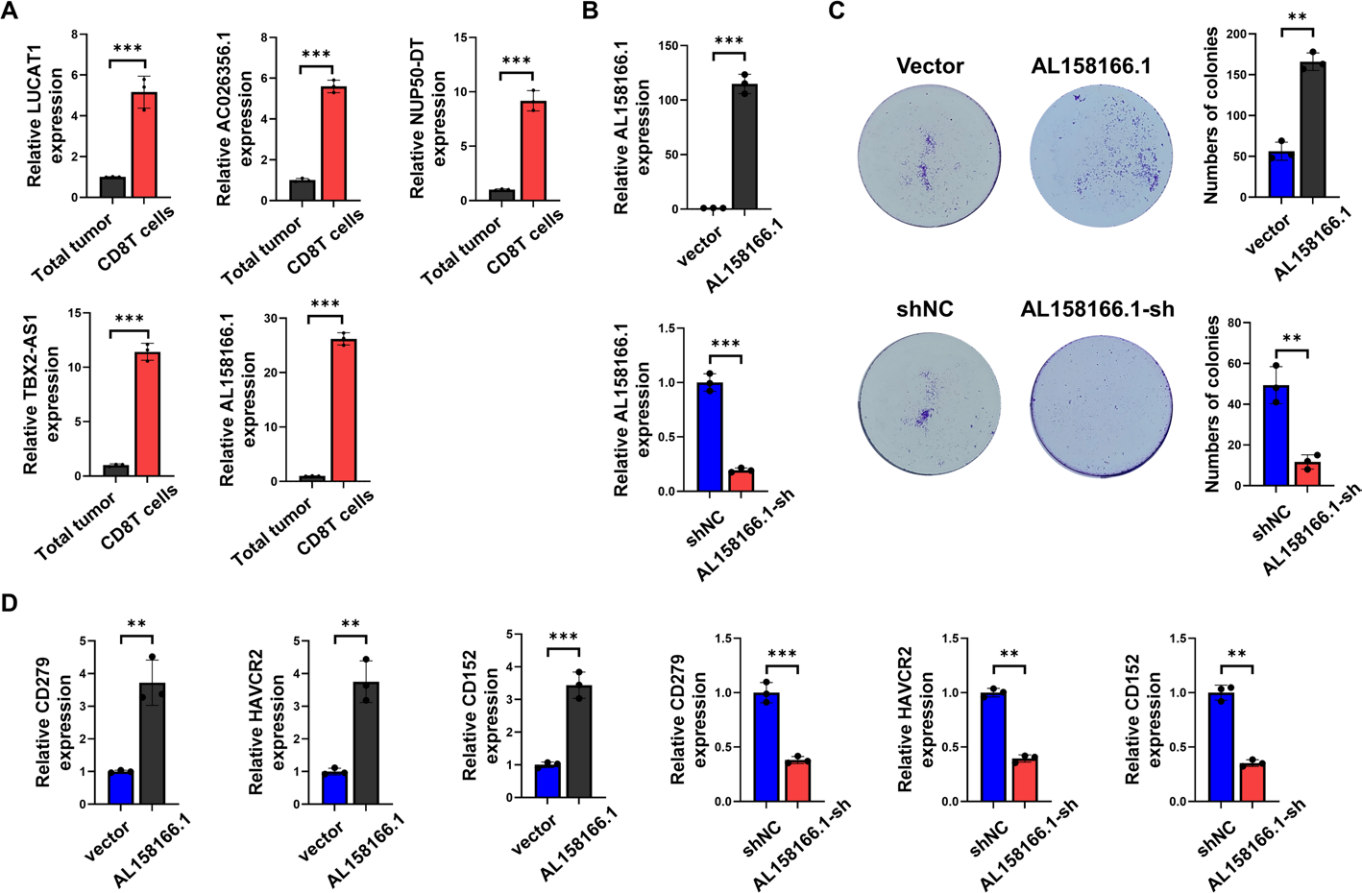
Functional validation of CD8⁺ Tex-associated lncRNA AL158166.1 in HCC. **A** Relative expression of five CD8⁺ Tex-associated lncRNAs (LUCAT1, AC026356.1, NUP50-DT, TBX2-AS1, and AL158166.1) in CD8⁺ T cells compared to total tumor tissue, as assessed by qRT-PCR. **B** qRT-PCR confirmation of AL158166.1 overexpression and knockdown in CD8⁺ T cells. **C** Colony formation assays showing that AL158166.1 overexpression enhances, while its knockdown reduces, the clonogenic potential of HCC cells co-cultured with CD8⁺ T cells. **D** Expression of exhaustion markers (CD279/PD-1, HAVCR2/TIM-3, and CD152/CTLA-4) in CD8⁺ T cells following AL158166.1 overexpression or knockdown, as measured by qRT-PCR

To validate the expression pattern of CD8⁺ Tex-associated lncRNAs in HCC, we isolated CD8⁺ T cells from dissociated human HCC tissues and performed qRT-PCR analysis. Among the five candidate lncRNAs identified from transcriptomic analysis—LUCAT1, AC026356.1, NUP50-DT, TBX2-AS1, and AL158166.1—all were significantly upregulated in CD8⁺ T cells compared to total tumor tissue, with AL158166.1 exhibiting the highest relative expression (~ 25-fold increase) (Fig. 9A). Given its predominant expression, AL158166.1 was selected for functional characterization. We established CD8⁺ T cells with AL158166.1 overexpression and knockdown, as confirmed by qRT-PCR (Fig. 9B). In a colony formation assay using co-cultures of CD8⁺ T cells and HCC cells, overexpression of AL158166.1 markedly enhanced tumor cell clonogenicity, whereas knockdown significantly impaired it (Fig. 9C), suggesting a functional role in promoting tumor growth.

To further explore its immunological impact, we assessed the expression of canonical exhaustion markers in AL158166.1-manipulated CD8⁺ T cells. Overexpression of AL158166.1 significantly elevated the expression of PD-1 (CD279), TIM-3 (HAVCR2), and CTLA-4 (CD152), indicating induction of an exhausted phenotype. Conversely, knockdown of AL158166.1 reduced the expression of these exhaustion-related genes (Fig. 9D). Collectively, these findings demonstrate that AL158166.1 is highly enriched in intratumoral CD8⁺ T cells and promotes T cell exhaustion and tumor progression, supporting its functional relevance as a key CD8⁺ Tex-associated lncRNA in HCC.

## Discussion

Compared to patients where tissue-resident memory T cells (TRM) dominate, those with CD8Tex dominance exhibit poorer survival outcomes. This association correlates with the molecular characteristics, metabolic profiles, and functional statuses of these cell populations. Moreover, enrichment of TEX remains independently prognostic beyond conventional factors such as disease staging, age, and tumor biomarkers. Lower TEX proportions are also linked to improved outcomes following checkpoint therapies [24]. Research by Shuai Wang et al. highlights DOCK2’s role in regulating CD8 + T cell infiltration in HCC, while cholesterol sulfate synthesized by tumor cell SULT2B1 promotes T cell exhaustion, thus impacting the efficacy of immunotherapy in HCC [25]. Another study demonstrates that itaconate derived from macrophages induces epigenetically mediated CD8 + T cell exhaustion, thereby promoting HCC progression [26]. Our findings corroborate that high expression of CD8Tex-related genes is consistently associated with poorer prognosis in HCC, affirming the oncogenic role of CD8Tex in disease progression. Additionally, we developed a prognostic model based on CD8Tex-related lncRNAs, which accurately predicts the prognosis of HCC patients based on calculated high-risk scores, further highlighting the prognostic estimation value of CD8Tex in HCC.

In contrast to other immune cells known for secreting various cytokines to resist tumors and recruit additional immune cells [27], the intercellular communication role of CD8Tex in HCC remains unclear. Our research reveals strong interactions between CD8Tex and CD8 + T cells, dendritic cells (DCs), and monocytes, while interactions with CD4T cells and B cells are comparatively weaker. Additionally, CD8Tex acts as a receptor regulated by families like HLA and CLEC2D from other cells and, when acting as a donor, regulates other cells via families such as PTPR and ITGB [28–30]. These findings suggest a pivotal role for CD8Tex in intercellular communication among immune cells in HCC. Furthermore, our study identifies specific immune pathways through which CD8Tex influences HCC progression, including significant downregulation of COAGULATION, potentially indicating CD8Tex’s specific immunological role in the liver [31]. Additionally, we found significant inhibition of important immune-related pathways like HALLMARK-ADIPOGENESIS, HALLMARK-ESTROGEN RESPONSE EARLY, and HALLMARK-FATTY ACID METABOLISM within CD8Tex. Further exploration of these pathways’ targets could unveil novel therapeutic strategies for targeted immunotherapy in the future.

Over the past decade, significant strides have been made in systemic therapies for advanced hepatocellular carcinoma (HCC). However, newly developed treatment strategies have not universally succeeded, often encountering therapy resistance in HCC patients [32]. Precision medicine represents a paradigm shift in cancer treatment, leveraging individual patients’ unique molecular characteristics to personalize therapy, aiming to maximize efficacy while minimizing side effects [33]. While precision therapies have shown remarkable success in various cancer types, their application in HCC is still emerging. Several potential biomarkers for predicting HCC treatment response have been identified, though few have been integrated into clinical practice [32]. Targeting lncRNAs could offer an economical, straightforward, and effective approach to treating HCC [15]. Additionally, as immunotherapy becomes a standard treatment, understanding the complex relationship between the tumor microenvironment (TME) and treatment outcomes becomes increasingly crucial [32, 34]. Our research has identified a novel molecular subtype of HCC based on CD8Tex-related lncRNAs, comprising five distinct classes. Our findings indicate that the C3 group exhibits minimal immune cell infiltration, categorized as an “immune desert” phenotype, while other groups may feature characteristics beyond immune factors. Using drug sensitivity analysis (IC50 values as indicators), we propose tailored targeted therapies for each subtype: C1 group may benefit from drugs like Elephantin, C2 group from drugs like ERK_6604, C3 group from drugs like XAV939, C4 group from drugs like VX-11, and C5 group from drugs like MG-132. This approach highlights the most suitable targeted therapies for different molecular subtypes of HCC, suggesting that clinical implementation of our subtype classification could improve prognostic outcomes for HCC patients.

Our experimental validation identified AL158166.1 as a key CD8⁺ Tex-associated lncRNA in HCC. Its overexpression in primary CD8⁺ T cells induced hallmark exhaustion markers (PD-1, TIM-3, CTLA-4), while knockdown reversed this phenotype, suggesting a causal role in promoting T cell dysfunction. Functionally, AL158166.1 impaired CD8⁺ T cell cytotoxicity and enhanced HCC cell proliferation in co-culture assays. These findings demonstrate that AL158166.1 is not only a biomarker of CD8⁺ T cell exhaustion but also a functional mediator of immune suppression and tumor progression, highlighting its potential as a therapeutic target in HCC.

This study indeed has some limitations [9]. This study has several limitations. First, although AL158166.1 was experimentally validated as a functional mediator of CD8⁺ T cell exhaustion, the underlying molecular mechanisms remain insufficiently characterized. Future investigations utilizing advanced techniques such as single-cell RNA sequencing and spatial transcriptomics in HCC tissues are warranted to delineate its regulatory pathways and cell-type-specific activity within the tumor microenvironment. Second, the associations between CD8⁺ Tex-related lncRNAs and clinical outcomes were primarily derived from retrospective correlation analyses. The absence of comprehensive lncRNA annotations and long-term follow-up data in current public datasets restricts the external validation of both our prognostic model and the proposed molecular subtypes. Prospective, well-annotated cohorts will be essential to establish their clinical utility. Third, although our molecular subtyping reveals distinct patterns of immune microenvironment heterogeneity in HCC, important clinical variables—such as prior treatments, underlying disease etiology, and coexisting conditions—were not fully captured and may confound the observed associations. Incorporating these parameters in future models will enhance interpretability and translational relevance. Finally, transcriptomic analyses provide only static snapshots of a dynamic tumor immune landscape. They may not adequately reflect temporal changes in CD8⁺ Tex states or lncRNA expression during disease progression or therapeutic intervention. Integrating longitudinal sampling strategies and multi-omics approaches will be critical to fully elucidate the biological and clinical significance of CD8⁺ Tex-related lncRNAs in HCC.

## Conclusion

Overall, we have elucidated the functions and immune characteristics of CD8Tex in HCC. Furthermore, we have established a robust prognostic model based on CD8Tex-associated lncRNAs and identified novel molecular subtypes, revealing immunological heterogeneity among HCC patients with different molecular classifications. Additionally, the novel molecular subtyping of HCC can guide clinical immune therapies for patients. Our study provides a solid theoretical foundation for clinical treatment of HCC and future development of targeted therapies.

## Supplementary Information

Supplementary material 1. Figure S1 Drug sensitivity analysis of different risk score groups. A) The box plot illustrates the sensitivity of different risk score groups to various drugs. *p < 0.05, **p < 0.01, ***p < 0.001.

Supplementary material 2. Figure S2 Drug sensitivity analysis of different HCC molecular subtypes. A) The box plot illustrates the sensitivity of different HCC molecular subtypes to various drugs. *p < 0.05, **p < 0.01, ***p < 0.001.

Supplementary material 3.

## Data Availability

The results published here are in whole based upon data generated by the TCGA Research Network: https://www.cancer.gov/tcga. All other relevant data can be found in the supplementary material and will be made available on request.
